# Influence of Dietary Supplementation with Yeast Culture and Microencapsulated Butyric Acid on Growth Performance, Carcass Traits, Gut Health, and Immune Status in Broilers

**DOI:** 10.3390/vetsci12040359

**Published:** 2025-04-12

**Authors:** Azhar Nazir, Ehsaan Ullah Khan, Muhammad Muneeb, Shafqat Nawaz Qaisrani, Saima Naveed, Sohail Ahmad, Rao Muhammad Kashif Yameen, Ali R. Al Sulaiman, Rashed A. Alhotan, Ala E. Abudabos

**Affiliations:** 1 Department of Animal Nutrition, Faculty of Animal Production and Technology, University of Veterinary and Animal Sciences, Lahore 54000, Pakistan; 2019-mphil-2243@uvas.edu.pk (A.N.); 2022-mphil-1331@uvas.edu.pk (M.M.); shafqat.qaisrani@uvas.edu.pk (S.N.Q.); saimamahad@uvas.edu.pk (S.N.); 2 Department of Poultry Production, Faculty of Animal Production and Technology, University of Veterinary and Animal Sciences, Lahore 54000, Pakistan; sohail.ahmad@uvas.edu.pk (S.A.); 2015-phd-1058@uvas.edu.pk (R.M.K.Y.); 3 Environmental Protection Technologies Institute, Sustainability and Environment Sector, King Abdulaziz City for Science and Technology, P.O. Box 6086, Riyadh 11442, Saudi Arabia; arsuliman@kacst.gov.sa; 4 Department of Animal Production, College of Food and Agriculture Sciences, King Saud University, P.O. Box 2460, Riyadh 11451, Saudi Arabia; ralhotan@ksu.edu.sa; 5 Department of Agriculture, School of Agriculture and Applied Sciences, Alcorn State University, 1000 ASU Drive, Lorman, MS 39096-7500, USA

**Keywords:** yeast culture, microencapsulated butyric acid, AGP alternative, broiler performance, gut health

## Abstract

The study aimed to examine the effects of dietary supplementation with microencapsulated butyric acid (EBA) and yeast culture (YC) in broiler diets. A total of 450 Ross-308 broiler chicks were selected and randomly allocated to five dietary treatments with six replicates (15 birds per replicate) in a complete block design. The experimental diets included the following treatments: (1) Negative control (NC) with basal diet without any additives. (2) Positive control (PC) with basal diet + 0.2 g/kg enramycin. (3) EBA, basal diet + 0.3 g/kg EBA. (4) YC, basal diet + 1 g/kg YC. (5) EBA+YC, basal diet + 0.3 g/kg EBA and 1 g/kg YC. The results indicated a non-significant effect on feed intake (FI) during the experiment periods. However, the EBA+YC treatment exhibited significantly increased body weight gain (BWG), better feed conversion ratio (FCR), and enhanced carcass traits (*p* < 0.05) compared to other treatments. A significant effect was observed for the immune organ weights and ND titters. Villus height (VH) and the ratio of villus height-to-crypt depth (VH: CD) were noted for EBA+YC across all other treatments. Ileal microbial analysis revealed a significantly lower count of *E. coli* and *Salmonella* in the ileal digesta of broiler chickens in the EBA+YC treatment compared to the NC group (*p* < 0.05). In conclusion, dietary supplementation with any supplement positively influences the broiler’s performance, carcass characteristics, gut health, and immune status over the NC group. More pronounced improvements were obtained from the EBA+YC group, indicating that EBA and YC had a synergistic effect on broilers.

## Simple Summary

Modern poultry farming encounters difficulties in enhancing growth, feed efficiency, and disease resistance while minimizing dependence on antibiotics. This study examined the potential benefits of integrating yeast culture, which boosts immune and nutrient absorption, and butyric acid, which aids in digestion, on broiler health and productivity. The study assessed the impact of various diets—having these supplements either alone or in combination—on gut health, growth, immunity, and meat quality. The findings indicated that the combination of both additives resulted in the most significant changes, including increased weight, improved feed conversion, enhanced immunological responses, and healthier digestive systems. Chickens consuming this diet exhibited enhanced nutrient absorption and diminished levels of pathogenic bacteria in their intestines, hence lowering the risk of illnesses. These findings demonstrate that employing such dietary interventions can improve poultry farming—benefiting through increased efficiency and reduced costs—while simultaneously promoting consumer health by decreasing the use of antibiotics in food production. This approach enhances sustainable poultry production, fostering improved animal welfare and environmental conservation.

## 1. Introduction

Gut health has been intensively researched, as the gut is deemed the primary location for nutrient digestion and absorption. Deteriorated gut health can decrease nutrient digestion and absorption, thus impacting poultry health and production performance [1]. Consequently, sustaining a healthy gut is essential for optimal well-being and productivity. Modern breeding plans and improved rearing systems have enabled broiler chickens to achieve about 2 kg body weight within 35 days [2]. With the advancement of the poultry industry and the need to ensure the sustainability of the food chain, there is a growing interest in achieving a better growth output with promising efficiency of feed utilization in broilers. The commercial broiler strains have the genetic potential of achieving higher growth rates at the least feed consumption, representing a better feed conversion ratio (FCR) [3]. However, this output capacity of broiler strains can only be put across by providing complete broiler rations that are nutritionally balanced and help the intestinal environment support maximum digestion and absorption of dietary nutrients [4]. The digestive system is widely recognized as vulnerable to numerous pathogens [5]. Consequently, a healthy gut environment for optimal production output can be better maintained with the help of different dietary additives [6]. Antibiotic growth promoters (AGPs) have been widely used in poultry production to enhance growth performance and maintain gut ecosystem balance, primarily due to their affordability and widespread availability [7]. However, in 2006, the European Union banned the use of AGPs in animal feeds due to their residual effects and transfer of drug-resistant genes [8]. Hence, the search for alternatives to AGPs is receiving significant interest [9]. These alternatives include but are not limited to prebiotics, probiotics, organic acids, yeasts, and enzymes [10]. Continuous research into an appropriate feed additive(s) that promotes broiler growth, is non-hazardous, and is cost-effective for optimizing gut health is vital.

Yeast, in many forms—i.e., fermented yeast, breweries or distillery yeast, and commercial yeast—is among the common feed additives in poultry diets [11]. Yeasts include both unicellular and multicellular species. Similarly, yeast size varies greatly, from 3–4 µm to over 40 µm [12]. Yeast is considered antagonistic to detrimental microbes, thereby causing a barrier effect and possibly helping protect the intestinal mucosa against assaulting germs. Furthermore, yeast fractions stimulate the host animal’s immune response [13]. The yeast culture (YC) is an important yeast product in the broiler industry. A YC can be defined as a distinct micro-ecological product consisting of a mixture of biomass with living yeast and other fermentation metabolites. The extracellular metabolites, like organic acids, alcohols, peptides, and esters, are the major constituents of YC [14]. The fermentation products of *Saccharomyces cerevisiae*, also called baker’s yeast, are widely used as YC [15]. YC was reported to provide protein, amino acids, trace essential minerals, and vitamins for broilers [12]. YC possesses both in vitro and in vivo antagonistic activity against the population of several pathogenic microbes [16]. YC supplementation significantly improves growth performance and cecal microbial community in broiler birds [17]. Other studies have also revealed valuable effects of dietary YC addition to poultry diets, such as improved performance [18], blood characteristics [19], humoral immunity response, and carcass characteristics [20].

Organic acids (OAs) are also effective additives in broiler feed and have been proven to improve broiler performance [21]. Organic acids are an extensive category of essential compounds used in the body’s basic metabolic processes [22]. The OAs are associated with several beneficial attributes in broilers: buffering the broiler’s diet, restricting harmful microorganisms in the intestine by altering the pH, raising the nutrients available from the diet, and improving immune responses in poultry [8]. Only the short-chain OAs (C_1_–C_7_) are specified for antimicrobial activity and may be produced through carbohydrate fermentation in the large intestine of broilers [22]. Among the OAs, the most common are short-chain fatty acids such as monocarboxylic acids, propionic, acetic, formic, and butyric acids, which may exist in their esterified form with calcium, sodium, and potassium salts [23]. Salts have advantages over acids because they are odorless and easy to handle in feed manufacturing [7]. Butyric acid (BA) is the most commonly used OA supplement in the broiler diet. Attributes for its use include better bioavailability, improved gut health, higher nutrient absorption, and enterocytes’ ease of absorbing more nutrients [24]. The intestinal villi can utilize BA as a readily available energy source that accelerates the differentiation and multiplication of villus cells [25]. This phenomenon improves mucosal nutrient absorption capacity and broiler feed efficiency [26]. Assembling of the host cell peptides is also stimulated by the presence of BA in the intestine, which further triggers cellular proliferation and encourages the development and repair of the gut [27]. It was proven that BA is un-dissociated at low pH and lipophilic, resulting in diffusion across the bacterial cell membranes, reducing the harmful microbial population [1]. Uncoated BA may hence get absorbed by the crop and proventriculus of the broiler, limiting its efficacy in the small intestine [28]. This issue, however, can be resolved through modern techniques like microencapsulation. The encapsulation of BA (EBA) through palm fat results in a slow release during transport through the intestinal tract [29] and ensures its beneficial utilization on the proper site of interest, that is, the duodenal area of the intestine [30].

As described, dietary supplementation with YC also alters the gut pH through its metabolites and microbiota modulation, i.e., secreting OAs like acetic acid and lactic acid. This drop in pH is detrimental to the survival of pathogens in the poultry gut [31]. Furthermore, yeast cells prevent pathogen colonization, modulate the host’s immune response, and maintain gut microbial homeostasis. The BA, like other OAs, also lowers the intestinal pH, improves nutrient absorption, and reduces pathogenic microorganisms in the gut [32]. Thus, their combined use can create synergy to boost broiler performance. The solitary effects of YC and OAs in broiler gut health optimization have been well documented in the existing literature. However, to the best of our knowledge, limited studies are available on their synergistic effects in broilers. Therefore, the current study aimed to compare the single and combined action of YC and EBA supplementation on broiler performance, carcass traits, immune response, and gut health. It was hypothesized that supplementing broiler diets with the combination of YC and EBA could better maintain the gut microbial balance and serve as a viable substitute for AGPs. This approach may have significant financial benefits for poultry producers by improving gut health, increasing feed efficiency, and reducing the medication costs of the flocks.

## 2. Materials and Methods

### 2.1. Ethical Approval

The experiment was carried out over 35 days at the Poultry Research and Training Centre, a floor-rearing broiler facility at the University of Veterinary and Animal Sciences (UVAS), Ravi Campus (C-block), Pattoki, Pakistan. All procedures conformed with the rules established by the Ethical Review Committee, UVAS, Lahore, Pakistan (Approval number: 196; Date: 16 March 2022).

### 2.2. Experimental Design

A total of 450 straight-run broiler chicks (Ross-308) with similar initial body weight were selected; then, chicks were divided into five treatments with six replicates of 15 birds each (90 chicks per treatment) in a completely randomized design. Treatment one was a basal diet without supplements (negative control (NC)). Treatment two served as the positive control (PC); the diet was provided with an AGP (EnraLiv^®^, Enramycin 4%) at 0.2 g/kg. The diet in treatment three was supplemented with EBA (ButiPEARL^®^, 45% Calcium Butyrate) at 0.3 g/kg. The diet in treatment four was supplemented with YC (GroPro^®^, Baker’s yeast derivative) at 1 g/kg. The diet in treatment five was supplemented with a combined EBA (0.3 g/kg) and YC (1 g/kg) (Table 1). The EBA and YC preparations used in this study were in powder form and added to the diet during manufacturing at the feed processing unit.

### 2.3. Bird Husbandry

The chicks were maintained according to the standard managemental practices of Ross-308 [33]. Briefly, before the placement of chicks, the house was preheated, and minimum ventilation was maintained. House temperature was initially maintained at 33 °C with the help of an electric brooder and decreased gradually by 2.8 °C per week until day 21. The relative humidity (RH) was stabilized at 65%. Later on, the environmental conditions (temperature, RH, and ventilation) were adjusted in line with the bird’s behavior and age. According to the ambient conditions during the experiment, the transitional ventilation system was mainly used after brooding. The house was kept under a 23 h light period throughout the experiment. Rice husk was used as litter material and evenly spread to a depth of approximately 2–5 cm on the floor. The bedding material was racked on alternative days to maintain its quality. Feed and fresh clean drinking water supply were given *ad libitum* throughout the experiment. The diets were prepared following Ross-308 nutrient specifications [34]. A broiler starter diet was fed from 1 to 21 d and a grower diet from 22 to 35 d (Table 2). All the feed ingredients and compounded diets were analyzed in the lab according to the standard protocols of AOAC.

### 2.4. Parameters Studied

Growth performance was measured as earlier explained [35]. Briefly, feed intake (FI), body weight gain (BWG), and feed conversion ratio (FCR) were determined. Mortality was taken daily and used to determine livability. Three birds per pen (18/treatment) were picked randomly at the end of the trial for further analysis. The sampled birds were individually weighed and were then slaughtered by exsanguination following electrical stunning. Subsequently, they were scalded, mechanically wet-plucked, processed, and eviscerated. The dressed weight was computed by dividing the eviscerated weight by the pre-slaughter BW and represented as a proportion [36]. On days 21 and 35, blood samples were collected from the wing veins of 18 birds per treatment for immune status. The samples were centrifuged at 2000 rpm to harvest serum. For Newcastle disease virus (NDV) antibody titer testing, the serum was aliquoted and stored at −20 °C in the experimental laboratory of the Department of Microbiology UVAS-Lahore. The antibody titers against NDV were determined using hemagglutination-inhibition (HI) [37]. The immune organ weights were also measured to assess the development of the immune system in birds. On day 35, 18 birds per treatment were sampled for microbial enumeration at the end of the experiment. One g digesta was taken from the ileum portion, transferred to sterile tubes containing PBS (phosphate buffer solution), and carefully taken to the laboratory for enumeration of the microbial population. Each sample (1 g) was tenfold serially diluted in a sterilized normal saline solution. Tenfold serially diluted samples were poured on Petri plates of selective media. *Salmonella* was grown on Brilliant Green agar media (Oxoid Basingstoke, UK), and *E. coli* on McConkey agar media (Oxoid, Basingstoke, UK). The colonies of these bacteria were quantified using a colony counter. Total colony-forming units were calculated by multiplying the mean number of colonies formed and the inverse of the dilution factor as previously outlined [38]. For studying the intestinal morphological changes, a 2 cm portion from the duodenum region (distal to the duodenal loop) was removed as the sample. The intestinal segments were washed and fixed in 10% formalin (48 h). The tissue samples were dehydrated in different dilutions of ethyl alcohol and then embedded in paraffin wax. Tissue sections (about 5 µm) were made using a microtome and mounted on a glass slide. A routine staining process was carried out by utilizing hematoxylin and eosin stains. The VH and crypt depth (CD) were observed through the 10X objective by using a light microscope. Measurements were taken and standardized with software (PixelPro^®^ 3.2^TM^, Labo America Inc., Fremont, NY, USA). A total of 10 well-oriented villi for each region were selected for measurement. Values obtained were averaged and used for statistical analysis.

### 2.5. Statistical Analysis

Pen replicates denoted the experimental unit for performance, while other analyses treated each bird as a separate unit. Data analysis was conducted using SAS 9.4 software (SAS Inc., Cary, NC, USA). Data underwent analysis through a one-way analysis of variance, with Tukey’s test used for multiple comparisons. Results were deemed significantly different at *p* values ≤ 0.05.

## 3. Results

### 3.1. Growth Performance

The results revealed a non-significant impact on the FI of birds in all the groups (*p* > 0.05). Feed intake during the starter (1–21 d) and grower phases (22–35 d) and in the overall period (1–35 d) was not affected by treatment. Improved BWG was reported during the starter, the grower, and the overall period due to feed supplementation compared to the un-supplemented diet, NC. The highest BWG was obtained from the group that had received EBA+YC compared to all other treatments (*p* < 0.001). The results showed that the EBA+YC group had 2.74%, 5.97%, 6.44%, and 15.29% higher BWG compared to EBA, PC, YC, and NC treatments, respectively. Starter, grower, and overall FCR were significantly improved when diets were supplemented compared to the un-supplemented group (NC). The best improvements with FCR were obtained from birds that received EBA+YC and EBA for the starter period; EBA+YC, EBA, and PC for the grower period; and EBA+YC and EBA for the cumulative period (*p* < 0.01, *p* < 0.001, *p* < 0.001, respectively). A substantial difference in the livability percentage of broiler chickens was observed. The NC possessed the lowest (*p* < 0.05) livability percentages in comparison to all other treatments (Table 3).

### 3.2. Carcass Traits

Table 4 presents the results of the treatment effects on the broiler’s carcass characteristics at d 35. The results revealed that the highest carcass yield was obtained from broilers that had received EBA+YC, which was significantly different from the NC and PC groups (*p* < 0.001). Insignificant differences were reported for EBA, YC, and EBA+YC (*p* > 0.05). Breast muscle yield followed the same trend as carcass yield; higher breast yield was obtained from EBA, YC, and EBA+YC (*p* < 0.001). Breast yield from the PC group was intermediate with no significant difference from the EBA+YC or NC groups. The NC group had the lowest breast muscle yield. Leg quarter yield showed no significant differences between treatments (*p* > 0.05). Similarly, heart, liver, and gizzard absolute weights showed no significant differences between all treatments (*p* > 0.05). The broilers supplemented with EBA+YC had considerably more bursal development when compared to the NC and PC groups (*p* < 0.05). The spleen weight was significantly higher for EBA+YC compared to the NC, PC, and YC (*p* < 0.01), but it was similar to the EBA group (Table 4).

### 3.3. Intestinal Morphology

A significant increase in VH, CD, and VH: CD was reported due to treatments (*p* < 0.001, 0.05, and 0.001, respectively). The highest VH (1776.2 µm) and VH: CD (7.3) were in broilers fed with the EBA+YC diet, which was higher than all other treatments except for VH: CD for the EBA group (Table 5). On the other hand, the highest CD (284.0) (*p* < 0.05) was observed in broilers with basal diet-fed without any supplementation (Table 5).

### 3.4. Immune Status

**Figure 1:**
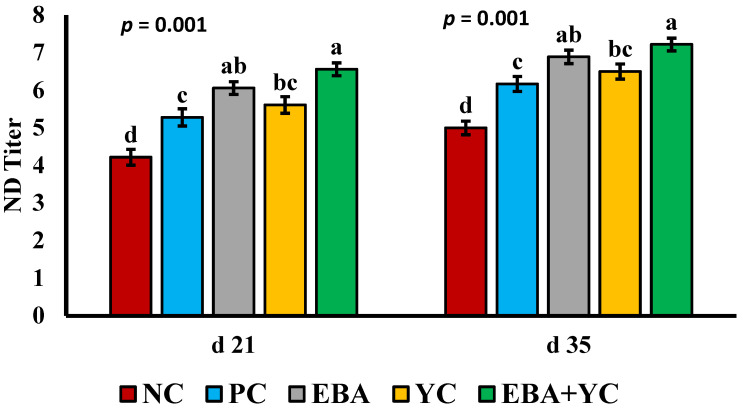
Effects of dietary treatments on the antibody titer against Newcastle disease in broiler chickens on days 21 and 35. ^a,b,c,d^ Means in the same row with different superscripts differ (*p* < 0.05).

The results showed a notable impact (*p* < 0.05) of added YC, EBA, and EBA+YC on the development and maturity of immune organs and NDV titers on d 21 and 35. The highest (*p* < 0.001) ND titers at d 21 and 35 were observed in the EBA+YC and EBA groups (Figure 1). Moreover, NDV titers were higher for the PC and YC groups compared to the NC (*p* < 0.001).

### 3.5. Intestinal Microbial Profile

**Figure 2:**
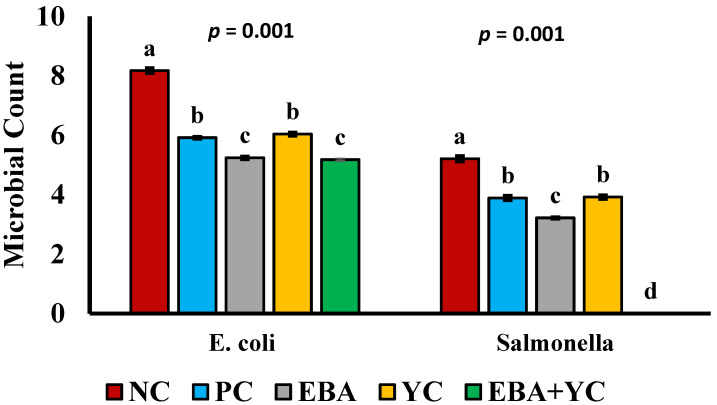
The effects of treatments on the ileal microbial count at 35 days (log_10_ CFU/g). ^a,b,c,d^ Means in the same row with different superscripts differ (*p* < 0.05).

The results regarding the influence of different treatments on intestinal microbial counts of broilers are described in Figure 2. The results indicated a significant (*p* < 0.001) decreased number of *E. coli* and *Salmonella* in the ileal digesta of broiler chickens for all supplemented groups compared to the NC. The EBA+YC group had the lowest *Salmonella* count compared to all other groups and the lowest *E. Coli* count compared to all groups except the EBA group (*p* < 0.001).

## 4. Discussion

In poultry production, sub-therapeutic antibiotics are frequently used to encourage growth and guard against bacterial infections. However, restrictions on their usage in animal production have prompted the development of alternatives to AGPs. The individual potentials of yeast-based products and organic acids are well described in the existing literature. However, their combined application has been limited, and only a few studies have examined the synergistic effects. Thus, this study aimed to assess the synergistic effects of YC and microencapsulated BA supplementation alone and in combination on performance, carcass traits, immune status, and gut health in broilers.

The non-significant difference in the FI among all the treatments obtained in this study is in alignment with the findings of Panda et al. [39], who concluded that supplementation of 0.05% butyric acid showed a non-significant difference in FI over the whole experimental period (1–35 d). Similarly, Zhen et al. [17] concluded that broiler FI was not affected by YC supplementation at different levels. The findings of this study are also consistent with those of Chand and Khan [40], who found that FI was unaffected by single-cell protein of yeast at the different levels during all broiler growth phases, while butyric acid increased the FI. This improvement in FI could be due to protection against gut pathogens through competitive exclusion, enhanced nutrient utilization, and improved growth feed efficiency by supplementing broiler diets with butyric acid [41].

The current study showed that treatments had a significant effect on the BWG of broilers during all phases. In line with a previous study, [38] found similar results of EBA on the BWG in broilers; they explained that organic acid’s low pH and antibacterial qualities prevent harmful gut microorganisms and lessen the production of detrimental byproducts; moreover, the improvement in the BWG of broilers was attributed to improving the digestibility of protein and energy. Furthermore, the supplementation of diets with propionic acid and butyric acid (0.2% and 0.3%) significantly increased the BWG of broiler chickens [42]. Additionally, the findings of our study regarding improved BWG concur with [43,44], who credited this increase in BWG to the optimized intestinal environment when supplementing broiler diets with butyric acid. In agreement with the results obtained herein, another group of researchers reported improved BWG when yeast was supplemented [45,46]. The yeast improved digestion, gut health, and nutrient absorption, resulting in better BWG. In the current study, there was an improvement in FCR due to EBA, YC, and their combination in all growth phases. Rationally, a higher BWG at a similar FI brought out the variation of FCR among the treatments. Organic acid supplementation at 2 g/kg [43,44] and encapsulated calcium butyrate at 0.2, 0.3, or 0.4 g/kg were reported to improve FCR [46]. The beneficial effects of EBA on intestinal microbiota, gut morphology, and digestive processes may be responsible for this increase in FCR [47]. Regarding the addition of YC in the broiler diet, our findings correlate with Alqahtani [10], who reported that YC supplementation at 0.25 g/kg can restore the growth performance of broiler chickens during a *C. perfringens* challenge, especially FCR. The YC contains various components, including β-glucans, which have been confirmed to positively influence the intestinal health of broiler chickens through diverse mechanisms.

High carcass and breast yields were obtained from EBA or YC independently, but the combination of the two additives (EBA+YC) further improved carcass yield. Butyrate was reported to improve carcass yield [46,47,48]. The results may be because of the ability of organic acids to enhance protein digestion, affect intestinal cell morphology, stimulate pancreatic secretions, act as a substrate for intermediate metabolism, improve nutrient retention, and control electrolyte balance in the intestine. These factors will provide the host animal with more nutrients for protein accretion. Other reports found that YC improved carcass and breast yield [49]. Contrary to the earlier findings, a non-significant difference in the carcass yield and the relative weight of giblets across all the treatments was noted by [19].

The immune system plays a significant role in poultry health regulation and disease prevention. In this study, the titer against NDV was higher for all treatments compared to the NC. This could be due to the reduced intestinal pH, increased intestinal integrity, and improved microbial balance in the intestine with OAs use, which leads to the enhancement of immune response. The results obtained herein aligned with those of [29,50], who concluded that sodium butyrate supplementation resulted in significantly higher NDV antibody titers in broilers and layers. Moreover, the current outcomes are consistent with those of Gao [51] and Muthusamy [52], who found that adding YC significantly improved the broilers’ antibody titer against NDV and improved the immune status in broilers. This may be explained by the beneficial effects of YC on preserving the physiological balance of immunopotent cells and, consequently, creating a robust immune system environment. This suggests that YC might increase antibody production by the humoral immune system. The intestinal mucosa is coated with more antibodies that shield villi from damage. It was suggested that the oligosaccharides found in YC walls might attach to viruses and function as vaccine adjuvants in YC-treated birds to raise antibody titers [53].

The findings of this study showed that treatments, especially EBA+YC, have a notable effect on the gut microbiota of broilers. In this study, the supplementation of EBA reduced *Salmonella* and *E. coli* counts in the small intestine. This might be explained by the lower gut pH caused by OAs, which is detrimental to the growth of acid-intolerant coliforms. Butyrate, among other SCFAs, has a higher efficacy against acid-intolerant coliforms like *Salmonella* and *E. coli*. The outcomes are similar to those of [39], who proposed that the *E. coli* population was significantly decreased in the gut with butyrate supplemented to broilers. When butyrate is supplemented to broilers, it rapidly releases sodium ions in the bird’s stomach; butyrate is swiftly transformed into the undissolved form known as butyric acid [54]. Since butyric acid is highly lipophilic and may permeate bacterial membranes, this form is the one that exhibits antimicrobial activity. Butyrate can prevent harmful bacteria from colonizing the lower portion of the intestinal system by blocking the production of genes that cause the invasion of the epithelial cells [55]. Findings of another study by Sun et al. [46] reported that YC supplementation to broilers significantly reduced the count of *E. coli* in comparison to the control group.

An essential indicator of intestinal health is the ratio of VL to CD. The results obtained for the intestinal morphometrics in this study agree with those of Ahsan et al. [2], who found that butyric acid supplementation significantly improved VH and VH: CD. The improvements in intestinal morphological characteristics are explained by butyrate absorption by the enterocytes and their function as an energy source, which could be related to the better gut health of birds. It can be inferred that the EBA and YC used have improved the intestinal absorptive area by enhancing VH. Increased VH and deeper crypts happen because of increased growth of the enterocytes and elongation of the villi. The ingestion of butyrate is known to alter the microstructure of intestines, and butyrate enhances enterocyte development, differentiation, and proliferation [27]. Similarly, the findings of another study [47] stated that sodium butyrate linearly improved the VH and VH: CD with increasing levels in the feed. Similarly, broilers fed EBA (0.3 g/kg) presented higher VH, whereas the CD was decreased compared to the control birds [46]. A high VH to CD ratio indicates a long villus in which the epithelium is sufficiently matured and functionally active, in combination with a shallow crypt with constant cell renewal. The effect on VH could be due to better pancreatic fluid secretion, as well as better digestion and absorption of dietary nutrients. Contrary to the findings of our study, Levy et al. [30] found non-significant variations in VH, CD, and VH: CD in the small intestine of broilers fed a diet having 0.3 g/kg EB compared to the NC. Considering the addition of YC in the broiler diet, our results are aligned with the results of different groups [12,15,45] who reported that broilers fed different levels of YC showed an increase in VH, CD, and VH: CD. In contrast to the above results, [56] concluded that YC supplementation to the broiler’s diet under heat stress resulted in a non-significant change in the amount of VH and CD in the small intestine.

## 5. Conclusions

The results of the present study demonstrated that EBA and YC supplements to the broiler’s diet independently enhanced the performance of broilers by improving BWG and FCR without negatively affecting FI. However, when these two additives were combined in treatment 5 (EBA+YC), there was a synergistic effect, and the positive response was more pronounced on performance. Additionally, this combination improved carcass traits, immune response, and intestinal morphometrics while effectively lowering the burden of harmful pathogenic bacteria in the ileum. The findings suggest that EBA and YC supplementation can be a good substitute for AGPs, promoting sustainable poultry production.

## Figures and Tables

**Table 1:** Experimental layout.

No.	Treatment	Phase	Total Number of Birds
		Starter Phase (1–21 d) and Grower Phase (22–35 d)	
1.	NC	Basal diet without any additive	No of treatments = 5 No of replicates = 6 Experimental units = 5 × 6 = 30 No of birds/replicate = 15 Total birds = 5 × 6 × 15 = 450 Ross-308 (Straight-run) Completely Randomized Design (CRD)
2.	PC	Basal diet +Enramycin at 0.2 g/kg	
3.	EBA	Basal diet + Microencapsulated Butyric acid at 0.3 g/kg	
4.	YC	Basal diet + Yeast culture at 1 g/kg	
5.	EBA+YC	Basal diet + Microencapsulated Butyric acid (0.3 g/kg) + Yeast Culture (1 g/kg)	

NC = Negative Control, without any additive; PC = Positive Control, Enramycin at 0.2 g/kg; EBA = Microencapsulated Butyric Acid at 0.3 g/kg. YC = Yeast Culture at 1 g/kg; EBA+YC = Combined Microencapsulated Butyric Acid and Yeast Culture at 0.3 g/kg and 1 g/kg, respectively. All the supplements were added at the mixer level and were part of pellets/crumbles.

**Table 2:** Basal diet feed formulation (as fed basis %).

Ingredients (%)	Starter (1–21 d)	Grower (22–35 d)
Corn	44.56	49.52
Soybean Meal	25.0	22.5
Canola Meal	11.0	11.0
Rice Polish	8.00	5.00
Corn Gluten Meal 30%	5.00	3.50
Canola Oil	2.96	5.25
L-Lysine HCL	0.45	0.35
DL-Methionine	0.21	0.18
Common Salt	0.50	0.50
Dicalcium phosphate	0.22	0.20
Limestone	2.00	2.00
Minerals and Vitamin Premix *	0.10	0.10
Total (%)	100	100
**Calculated Nutrients**		
Metabolizable energy (ME, Kcal/kg)	3000	3200
Crude protein, %	23.00	21.5
Ether extract, %	5.93	6.92
Crude fiber, %	4.63	4.63
Calcium %	0.96	0.87
Phosphorus %	0.48	0.43
Dig-Lysine %	1.28	1.15
Dig-Methionine %	0.51	1.47
Dig-Threonine %	0.86	0.77
**Analyzed Nutrients (%)**		
Crude Protein	22.96	20.99
Crude fiber	4.61	4.64
Ether extract	5.91	6.93
Crude ash	1.63	1.63
Calcium	0.96	0.87
Phosphorus	0.47	0.44

* Fe (sulfate), 40 mg; I (iodide), 0.15 mg; Cu (sulfate), 16 mg; Se (selenate), 0.3 mg; Mn (oxide and sulfate), 120 mg; Zn (oxide and sulfate), 100 mg; mineral oil, 3.75 mg; and cereal-based carrier, 128 mg; vit. A, 12,000 IU; vit. D3, 5000 IU; vit. K, 3 mg; vit. E, 75 mg; vit. B2, 8 mg; vit. B3, 55 mg; vit. B1, 3 mg; vit. B6, 5 mg; vit. B12, 16 µg; vit. B9, 2 mg; vit. B5, 13 mg; biotin, 200 µg; mineral oil, 2.5 mg; and cereal-based carrier, 149 mg.

**Table 3:** Effects of different treatments on growth performance of broilers.

Parameter	Treatments					*p*-Value
	NC	PC	EBA	YC	EBA+YC	
Initial BW (g)	45.0 ± 0.02	45.0 ± 0.02	45.0 ± 0.02	45.0 ± 0.02	45.0 ± 0.02	0.790
**d 1–21**						
BW (g)	844.1 ^d^ ± 6.82	872.5 ^c^ ± 6.98	899.8 ^b^ ± 7.09	875.6 ^c^ ± 7.28	922.5 ^a^ ± 7.33	0.001
BWG (g)	799.1 ^d^ ± 6.81	827.5 ^c^ ± 6.99	854.8 ^b^ ± 7.07	830.6 ^c^ ± 7.28	877.5 ^a^ ± 7.34	0.001
FI (g)	1132.7 ± 3.62	1149.7 ± 8.90	1150.7 ± 3.55	1139.4 ± 7.55	1153.3 ± 10.96	0.277
FCR	1.417 ^a^ ± 0.01	1.389 ^ab^ ± 0.02	1.346 ^bc^ ± 0.01	1.372 ^ab^ ± 0.02	1.314 ^c^ ± 0.02	0.007
**d 22–35**						
BW (g)	1873.1 ^d^ ± 15.84	2033.8 ^c^ ± 16.20	2096.3 ^b^ ± 16.88	2025.0 ^c^ ± 15.94	2152.5 ^a^ ± 17.21	0.001
BWG (g)	1029.0 ^d^ ±11.33	1161.3 ^c^ ± 9.33	1196.5 ^b^ ± 10.46	1149.4 ^c^ ± 10.37	1230.0 ^a^ ± 10.66	0.001
FI (g)	2089.3 ± 10.35	2097.0 ± 20.02	2100.9 ± 3.63	2098.8 ± 24.62	2117.7 ± 27.38	0.882
FCR	2.030 ^a^ ± 0.01	1.806 ^bc^ ± 0.02	1.756 ^bc^ ± 0.02	1.826 ^b^ ± 0.04	1.722 ^c^ ± 0.04	0.001
**d 1–35**						
BWG (g)	1828.1 ^d^ ± 15.82	1988.8 ^c^ ± 16.21	2051.3 ^b^ ± 16.86	1980.0 ^c^ ± 15.95	2107.5 ^a^ ± 17.22	0.001
FI (g)	3222.0 ± 12.61	3246.6 ± 26.62	3251.6 ± 3.83	3238.2 ± 31.29	3271.0 ± 38.21	0.745
FCR	1.763 ^a^ ± 0.01	1.632 ^b^ ± 0.02	1.585 ^bc^ ± 0.01	1.635 ^b^ ± 0.03	1.552 ^c^ ± 0.03	0.001
Liv (%)	92.5 ^b^ ± 0.67	94.3 ^ab^ ± 0.92	95.6 ^ab^ ± 0.86	93.5 ^ab^ ± 1.11	96.1 ^a^ ± 1.40	0.113

^a,b,c,d^ Means in the same row with different superscripts differ (*p* < 0.05). NC = Negative Control, without any additive; PC = Positive Control, Enramycin at 0.2 g/kg; EBA = Microencapsulated Butyric Acid at 0.3 g/kg; YC = Yeast Culture at 1 g/kg; EBA+YC = Combined Microencapsulated Butyric Acid & Yeast Culture at 0.3 g/kg and at 1 g/kg, respectively; BW = body weight; BWG = body weight gain; FI = feed intake; FCR = feed conversion ratio; Liv = livability.

**Table 4:** Carcass yield and some internal organ weights as a percentage of pre-slaughter weight in broiler chickens at 35 d.

Parameter	Treatments					*p*-Value
	NC	PC	EBA	YC	EBA+YC	
Carcass yield (%)	68.7 ^c^ ± 0.40	70.0 ^b^ ± 0.34	70.6 ^ab^ ± 0.33	70.5 ^ab^ ± 0.34	71.3 ^a^ ± 0.34	0.001
Leg quarter (%)	23.4 ± 0.27	23.5 ± 0.26	23.7 ± 0.22	23.6 ± 0.24	24.0 ± 0.23	0.150
Breast weight (%)	24.7 ^b^ ± 0.24	25.5 ^ab^ ± 0.25	26.3 ^a^ ± 0.25	26.5 ^a^ ± 0.23	26.5 ^a^ ± 0.22	0.001
Heart weight (g)	8.5 ± 0.14	8.7 ± 0.16	8.8 ± 0.15	8.8 ± 0.07	8.9 ± 0.11	0.338
Liver weight (g)	41.7 ± 0.28	41.8 ± 0.41	42.0 ± 0.41	42.1 ± 0.25	42.2 ± 0.27	0.841
Gizzard weight (g)	33.5 ± 0.55	33.7 ± 0.21	33.9 ± 0.19	33.8 ± 0.23	34.0 ± 0.20	0.796
Spleen (g)	2.31 ^c^ ± 0.07	2.56 ^bc^ ± 0.11	2.82 ^ab^ ± 0.10	2.63 ^bc^ ± 0.15	3.00 ^a^ ± 0.10	0.003
Bursa (g)	1.79 ^c^ ± 0.10	1.91 ^bc^ ± 0.11	2.15 ^ab^ ± 0.09	2.05 ^abc^ ± 0.11	2.29 ^a^ ± 0.08	0.043

^a,b,c^ Means in the same row with different superscripts differ (*p* < 0.05). NC = Negative Control, without any additive; PC = Positive Control, Enramycin at 0.2 g/kg; EBA = Microencapsulated Butyric Acid at 0.3 g/kg; YC = Yeast Culture at 1 g/kg; EBA+YC = Combined Microencapsulated Butyric Acid & Yeast Culture at 0.3 g/kg and at 1 g/kg, respectively.

**Table 5:** Effects of different treatments on intestinal morphology of commercial broilers (35 d).

Parameter	Treatments					*p*-Value
	NC	PC	EBA	YC	EBA+YC	
VH (µm)	1126.8 ^d^ ± 33.09	1393.8 ^c^ ± 32.83	1568.0 ^b^ ± 35.40	1495.8 ^b^ ± 31.29	1776.2 ^a^ ± 33.26	0.001
CD (µm)	284.0 ^a^ ± 21.07	283.8 ^a^ ± 18.53	235.2 ^b^ ± 7.76	277.5 ^ab^ ± 10.87	243.7 ^ab^ ± 6.33	0.050
VH: CD	4.09 ^c^ ± 0.33	5.03 ^b^ ± 0.38	6.69 ^a^ ± 0.20	5.42 ^b^ ± 0.20	7.30 ^a^ ± 0.14	0.001

^a,b,c,d^ Means in the same row with different superscripts differ (*p* < 0.05). NC = Negative Control, without any additive; PC = Positive Control, Enramycin at 0.2 g/kg; EBA = Microencapsulated Butyric Acid at 0.3 g/kg; YC = Yeast Culture at 1 g/kg; EBA+YC = Combined Microencapsulated Butyric Acid & Yeast Culture at 0.3 g/kg and at 1 g/kg, respectively; VH = villus height; CD = crypt depth; VH: CD = Villus height into crypt depth ratio.

## Data Availability

The authors declare that all the data and materials used in this study comply with field standards and are available on demand.
